# Total hip arthroplasty-related osteogenic osteosarcoma: case report and review of the literature

**DOI:** 10.1186/s40001-016-0203-3

**Published:** 2016-03-01

**Authors:** Rajko Kavalar, Samo K. Fokter, Janez Lamovec

**Affiliations:** Department of Pathology, University Medical Centre Maribor, Ljubljanska ulica 5, 2000 Maribor, Slovenia; Department of Orthopedics, University Medical Centre Maribor, Maribor, Slovenia; Department of Pathology, Institute of Oncology, Ljubljana, Slovenia

**Keywords:** Total hip arthroplasty, Loosened prosthesis, Osteogenic osteosarcoma

## Abstract

**Background:**

Orthopedic implant-related sarcoma is an exceedingly rare, but a known complication of total hip arthroplasty (THA).

**Case presentation:**

The authors describe clinical and radiologic features, histologic appearance, and treatment of osteogenic osteosarcoma located in the proximal femoral diaphysis associated with an unstable femoral prosthesis following THA in a 65-year-old male patient. The patient with HLA-B27 positive ankylosing spondylitis underwent arthroplasty 15 years ago.

**Conclusions:**

The neoplastic process may be considered as an extraordinary complication of THA and might just be coincidental or the result of some derangement of the healing process in host tissue with no definitely proven hypothesis that the implants or their by-products are carcinogenic. The soluble chemical substances from the implanted prosthetic material are, at least in animals, suspected to play a vital role in the pathogenesis of the neoplastic transformation of the bone tissue. The presented case shall alert orthopedic surgeons to clinical, radiologic, and macroscopic similarities between a malignant tumor and benign lesions caused by wear debris at THA sites. At the examination of plane X-rays of patients with THA loosening, the differential diagnosis should always include osteogenic sarcoma, as well. To our knowledge, there have been only nine cases of THA-related osteogenic osteosarcomas described in the English-language literature.

## Background

The exact etiopathogenesis of the osteosarcoma is poorly understood. Most of them arise de novo, but some of them appear in the setting of some premalignant conditions. Among them are congenital syndromes associated with osteosarcoma (familial retinoblastoma, Bloom syndrome, familial Paget disease, Li-Fraumeni syndrome, Werner syndrome, Rothmund–Thomson syndrome), Paget disease, giant cell tumor, osteoblastoma, and fibrous dysplasia [1].

Radiation exposure, chronic osteomyelitis, and metal endoprosthesis implantation are also well-known predisposing factors for osteosarcoma [2–6].

Sarcoma at the site of the metal foreign bodies was reported in the Western literature as early as 1888 [7]. However, the malignancies in the vicinity of the total hip arthroplasty (THA) are extremely rare. In the 30-year-long period—from 1974 to 2003, 46 different malignant tumors at the site of the THA were reported in the English-language literature and among them only 9 were osteosarcomas [3, 5, 6, 8–20].

Herewith, we describe a case of a THA-related osteogenic osteosarcoma.

## Case report

**Fig. 1:**
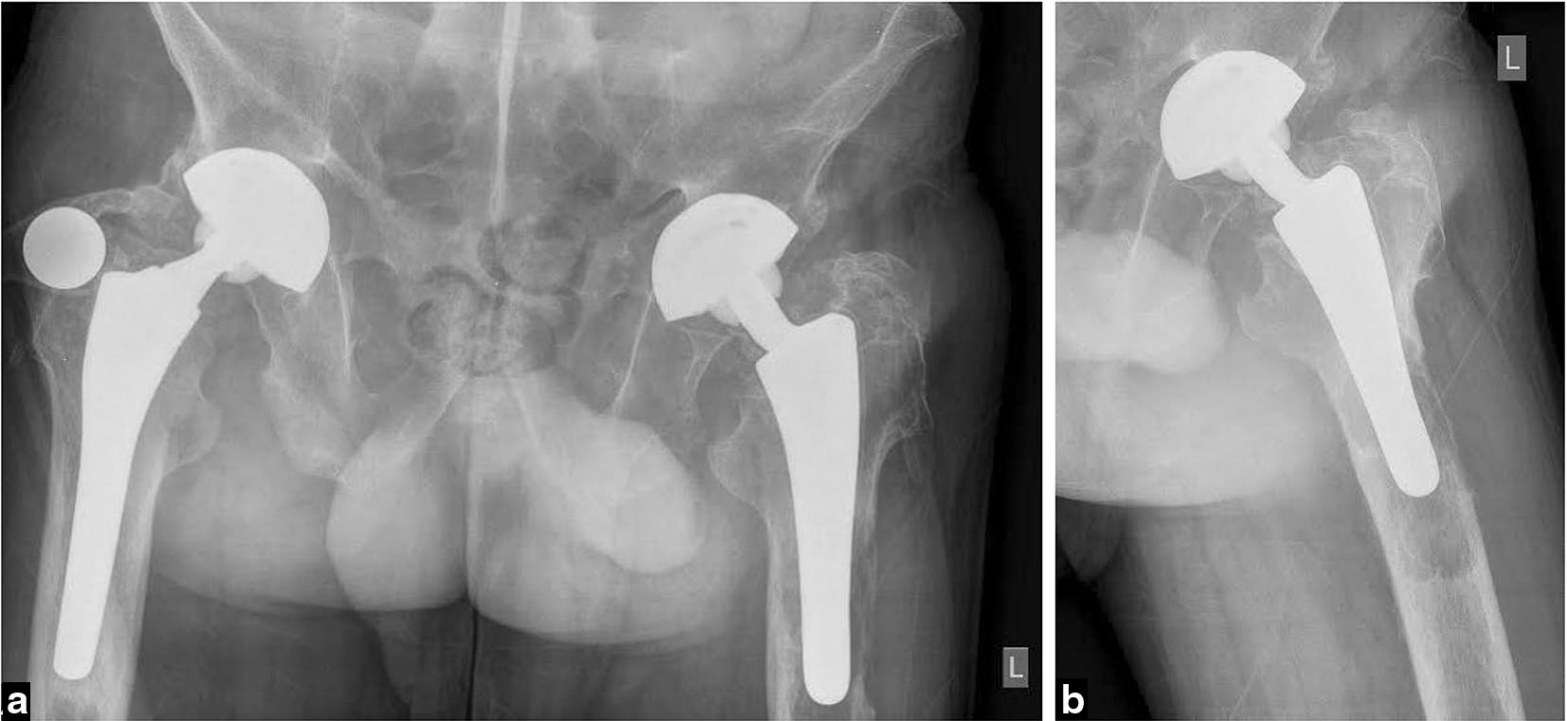
**a** Anteroposterior radiograph of the pelvis with both THA inserted. Note signs of femoral stem loosening on the left side. **b** Left hip lateral radiograph with osteolytic zone around the distal femoral stem

A 65-year-old Caucasian male was admitted to our hospital with a history of slowly developing pain in his left thigh in April 2015. The patient has undergone a THA of his left and right hip, respectively, due to HLA-B27 positive ankylosing spondylitis (AS) fifteen and twelve years prior to this presentation. Clinical examination disclosed a typical stiff spine and a pain-free right hip with excellent range of motion (ROM). Left hip was painful and has reduced ROM. There was no sign of palpable soft-tissue mass or enlarged inguinal and pelvic nodes. Pelvic and hip X-rays showed a radiolucent osteolytic zone around the tip of the uncemented left femoral stem consistent with aseptic loosening of prosthesis (Fig. 1).

Chest X-ray and blood tests were normal. According to these findings, the patient was scheduled for regular revision and replacement of the left THA. The prosthesis used in primary THA was an anatomically shaped uncemented model, made of Titanium Aluminum Vanadium (Ti6Al4V) Alloy (Ti, Al-6 %, V-4 %) proximally coated with hydroxyapatite.

During the revision procedure, the femoral stem was found loose and the acetabular component was well fixed. However, there were signs of wear and tear at the modular head and acetabular inlay. It was decided to replace all components of the primary arthroplasty. Routine tissue samples were collected for microbiological analysis and histologic examination from several different locations. The femoral bone was thinned and the soft tissue around femoral shaft of the loosened prosthesis was not suspicious for tumor. Frozen section of the tissue around the loosened femoral stem was not performed.

**Fig. 2:**
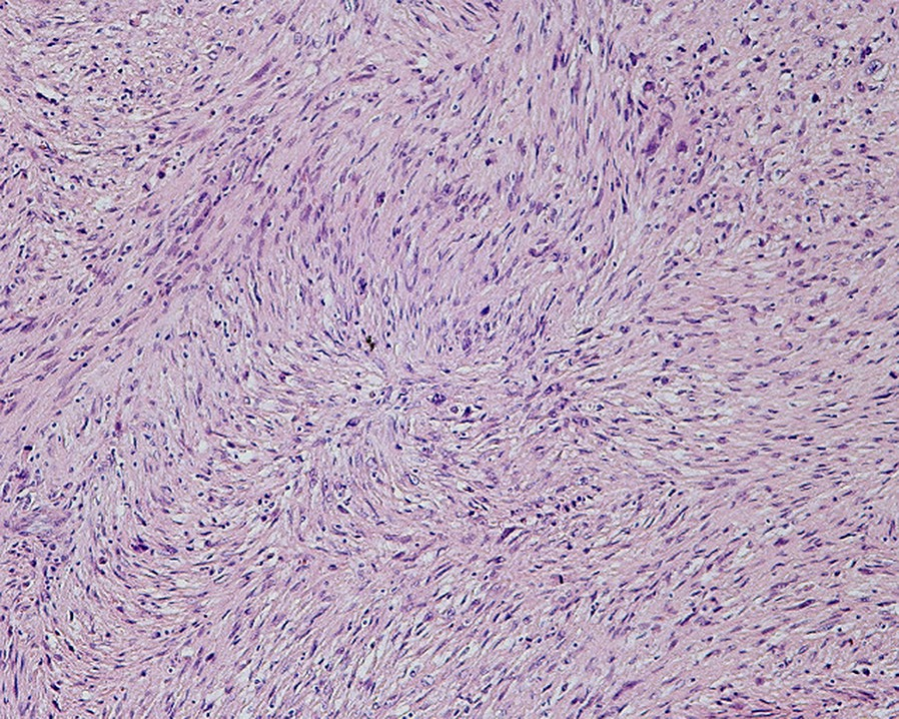
Malignant spindle cells arrangement in poorly formed fascicular pattern in fibroblastic part of the tumor (HE, 100×)

Several small tissue samples from different locations (labeled as pseudocapsule, bone, tissue from the femoral canal) were submitted for histopathologic examination. In some of the samples, rare thin normal bony trabeculae were found; in most samples the submitted tissue was hypercellular, fibroblastic, and arranged in poorly formed fascicles (Fig. 2).

**Fig. 3:**
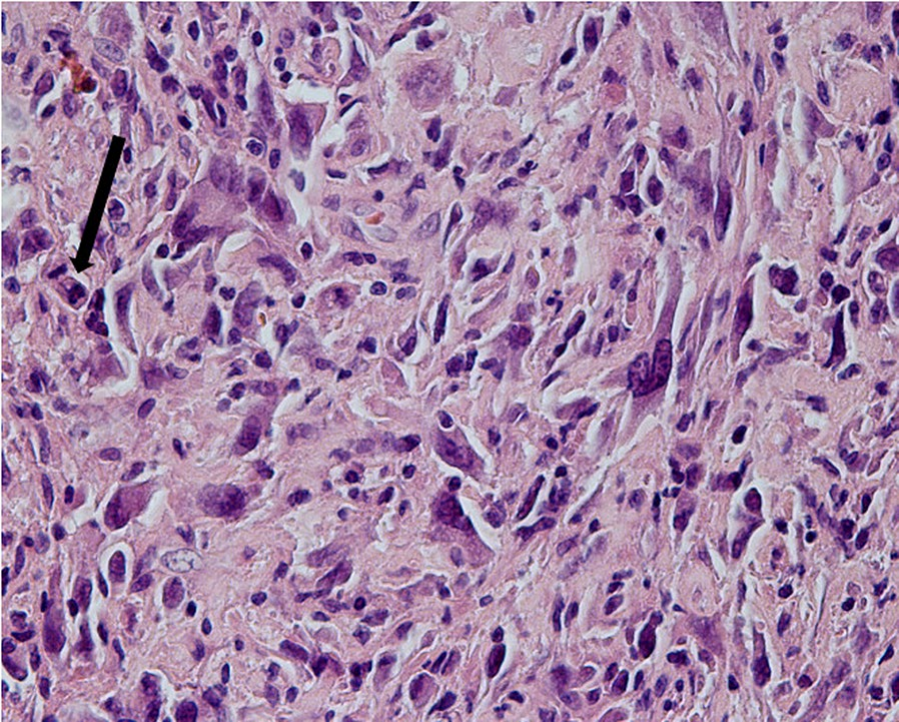
Highly pleomorphic cells; some multinucleated with minimal deposition of intercellular osteoid in the top of the picture. Mitotic figure is present—see *arrow* (HE, 200×)

More collagenized tissue revealed a less dense cellularity. The cells were focally pleomorphic and haphazardly arranged. Necroses were not found. Mitotic figures were present in hypercellular spindle- and pleomorphic-cell areas (Fig. 3).

**Fig. 4:**
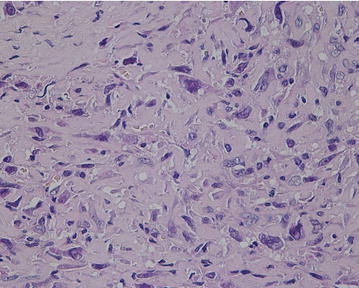
Non-mineralized eosinophilic osteoid between tumor cells in osteoblastic focus (HE, 200×)

In less than 10 % of examined tissue, a lace-like eosinophilic osteoid without signs of mineralization or ossification was seen among pleomorphic cells (Fig. 4).

**Fig. 5:**
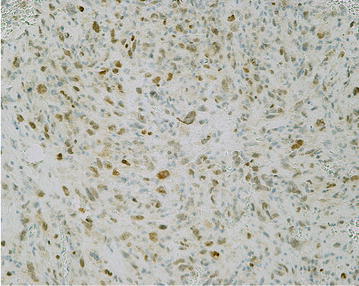
Most of the cells in osteoblastic foci were SATB2 immunoreactive (SATB2, 100×)

All tumor cells in fibroblastic areas were immunohistochemically diffusely positive to NSE, CD99, smooth muscle actin, calponin, focally for CKAE1/AE3, and calretinin. Only few isolated cells were positive for EMA, β-catenin, E-cadherin, S-100 protein, caldesmon, CD56 NCAM, Bcl-2, and CD117. All cells were negative for estrogen and progesterone receptors, CD34, and desmin. Predominantly in osteoblastic foci, the cells were mostly SATB2 positive (Fig. 5).

The so-called arthroplasty effect was not present in or near the tumor. According to morphology and immunohistochemistry, the lesion was recognized as a secondary, orthopedic implant-related conventional osteogenic osteosarcoma, a fibroblastic type with rare osteoblastic foci, without lymphovascular invasion. All microbiology samples were sterile.

**Fig. 6:**
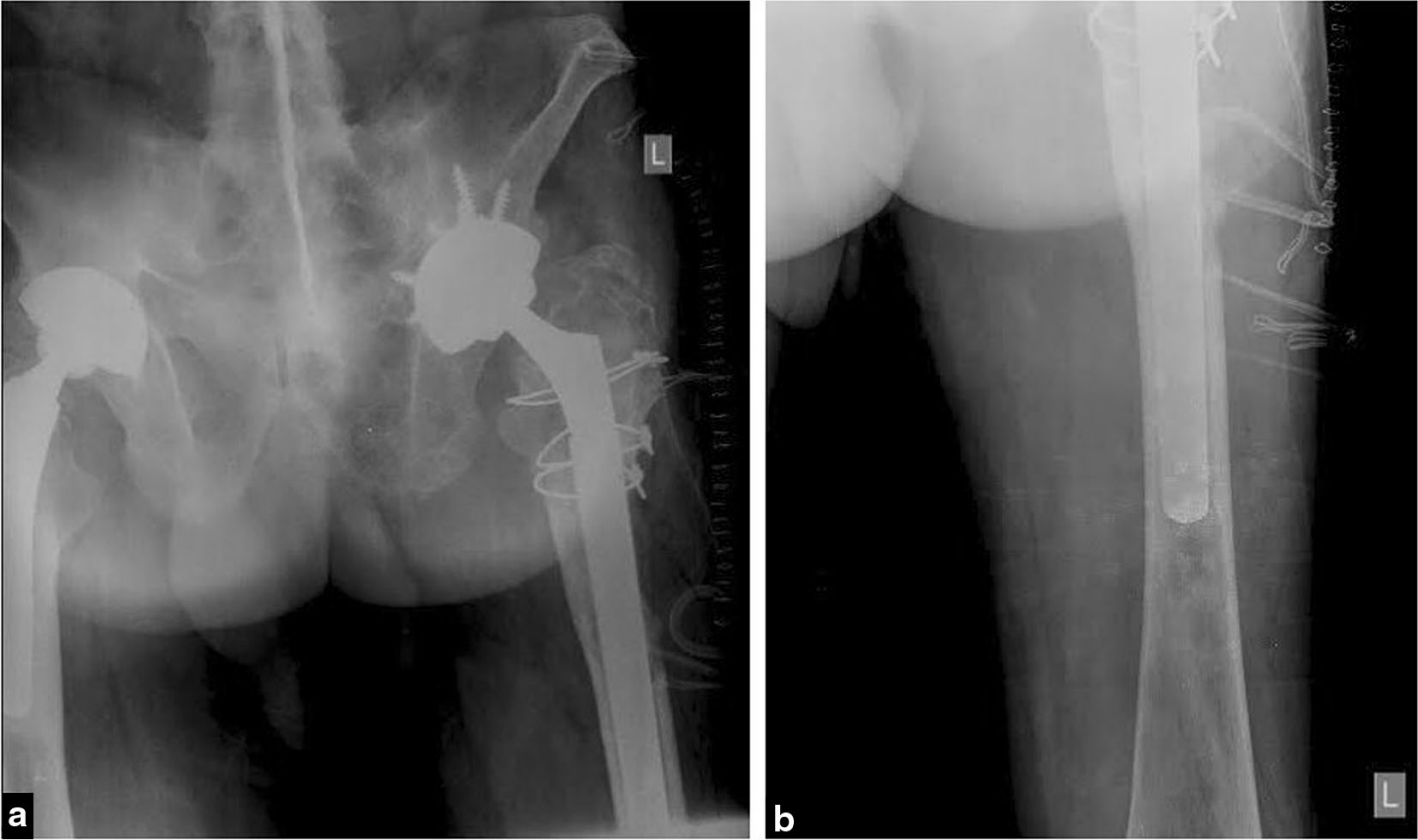
**a** Anteroposterior radiograph of the pelvis taken after revision of THA on the left side. **b** Anteroposterior radiograph of the femur showing tip of long revision femoral stem

Unaware of the histological diagnosis during the operation, the orthopedic surgeon did not perform a radical femoral resection. Instead, an uncemented revision total hip prosthesis with long femoral stem was inserted (Fig. 6).

The postoperative course was uneventful and the patient was discharged on day 6 postoperatively. Regarding the histopathological diagnosis, the patient was readmitted for computed tomography of the chest revealing two possible lung metastases and suspicious lesions in the 4th, 9th, and 10th thoracic vertebrae. Since the tumor has not been radically excised and a possible metastatic dissemination was suspected, the patient was treated with adjuvant chemotherapy (cisplatin, doxorubicin, and Lonquest to reduce neutropenia); 6 months after the diagnosis was established, he is well and shows no signs of local recurrence or systemic progression.

## Discussion

Artificial orthopedic hardware made of metals, ceramics, or plastic materials has been used for decades to repair the function of poorly functioning and painful joints. Only in 1996, over 140,000 THA were performed in the USA [21]. All those biomaterials are generally believed to be nontoxic. However, some of the constituents have been shown to promote neoplasia in the animal studies [22–27]. Sinibaldi reported an association of malignancies and the orthopedic implants in pets and Murphy described a case of osteosarcoma following THA in a dog [28, 29]. Foreign bodies of metallic cobalt, metallic nickel, and alloy powder containing 66–67 % of nickel, 13–16 % of chromium, and 7 % of iron have been classified as possible carcinogens to humans. Contrary, implanted foreign bodies made of metallic chromium or titanium and of cobalt- and/or titanium-based alloys, stainless steel, and organic polymeric materials are not believed to be carcinogenic to humans [30]. High concentration of prosthetic metal in synovial fluid and capsular tissue around the loosened prostheses are believed to be the result of continuous liberation of the metal and plastic particles from the constant rubbing of the artificial joint’s surfaces [31, 32].

Our review of the literature identified nine previously described cases; ours would be the tenth case of osteosarcoma associated with THA. Data about prosthesis type, material the prosthesis was made of, its mode of fixation, preoperative diagnosis, latent period from arthroplasty, age and sex of the patient, involved tissue, treatment and outcome are summarized in Table 1 (some data are missing). In four patients, the prosthesis was anchored with cement and in four patients (including ours) the prosthesis was uncemented. All patients were exposed to known common metal alloys used for THA (3 to CoCr, 3 to Ti alloy, 2 to stainless steel). The mean patient’s age at the time of diagnosis was 65.8 years and ranged from 54 to 75 years. 6 patients were female, and four were male. Six patients underwent THA for osteoarthritis (OA), one had a hip dysplasia with secondary OA, one had radiation-induced necrosis of the femoral head, and one had AS. In all ten patients, the bone and the surrounding soft tissue was involved by the sarcoma. The time interval between THA and diagnosis of the tumor ranged from 6 months to 15 years (mean 6.4 years for all ten patients).

**Table 1:** Total hip arthroplasty-related osteosarcoma

References	Age (year)/sex	Preoperative diagnosis	Prosthesis	Stem-head alloy; cup; fixation type;	Time lap (year)	Symptoms	Involvement; treatment; outcome
Penman and Ring [5]	75/F	OA	Ring	CoCr; CoCr; uncemented	5.0	Gross edema of the hip region; reduced ROM	Bone/soft tissue; none; death, 19 day; renal mets
Rushford [3]	54/F	Radiation necrosis of femoral head	McKee-Farrar	CoCr; CoCr; cemented	0.5	Increasing pain in both hips, more marked on the left side	Bone/soft tissue; Girdlestone, Tu not treated; death, 9 month; lung mets
Brien et al. [6]	60/F	Hip dysplasia, secondary OA	Chamley	Stainless steel; PE; cemented	8.0	Increasing pain in the thigh, buttock; palpable mass around the adductor muscle	Bone/soft tissue; chemotherapy, en block resection, with custom made prosthesis; ND
Martin et al. [20]	66/F	OA	Chamley-Mueller	CoCR; PE; cemented	10.5	Pain in the right hip; reduced ROM	Bone/soft tissue; hip-exarticulation; death, 8 month; lung mets
Prasad et al. [16]	70/M	OA	Howse II	TiAlV; PE/Titanium; uncemented	6.5	Pain, swelling of the hip	Bone/soft tissue; revision of the prosthesis; death, 7 week; lung mets
Adams et al. [17]	62/M	OA	Harris-Galante	Titanium stem, CoCr modular head; titanium shell with PE inlay; uncemented	3.0	Pain, stiffness and swelling of the hip	Bone/soft tissue; wide amputation; ND
Keel et al. [18]	73/F	OA	ND	ND; ND; ND	2.5	Pain	None/soft tissue; radiation; death, 1 year; lung mets
Keel et al. [18]	68/M	OA	ND	TiAlV; ND; ND	3.0	Pain, stiffness and swelling of the hip	Bone/soft tissue; hemipelvectomy; death, 6 month; lung mets
Lamovec et al. [13]	65/F	ND	Chamley-Mueller	Stainless steel; PE; cemented	10.0	Pain, reduced ROM	Bone/soft tissue; radiotherapy; death during treatment; lung mets
Kavalar et al. (Present case)	65/M	AS	Anatomic modular stem; press-fit acetabular cup	TiAlV stem with aluminal ceramic head; TiAlV shell with aluminal ceramic inlay; uncemented	15.0	Pain, reduced ROM	Bone/soft tissue; revision of total hip prosthesis; alive, 6 month; lung, ribs mets

*OA* osteoarthritis, *AS* ankylosing spondylitis, *PE* polyethylene, *ROM* range of motion, *ND* no data

The leading symptoms of the affected hips were increasing pain, stiffness, swelling, and reduced ROM. Lung metastases were present in seven patients and renal metastases in one patient. The prognosis of the patients with THA-related osteosarcomas was poor: the mean survival time from the diagnosis to death was 6 months, ranged from 9 days to 1 year (for six patients).

An unusual aspect of the presented case is that the patient had his bilateral THA performed 12 and 15 years previously—much longer than the average interval between THA and diagnosis of the tumor reported in reviewed cases. Both implanted THA were of the same type and mode of fixation indicating the same influence of the implanted material or their by-products on the bone and soft tissue of both hips. And yet, osteosarcoma has emerged on one side only, which casts some doubt on the role of the former factors in its pathogenesis.

## Conclusions

We present a case of one-side THA-related osteogenic osteosarcoma in a 65-year-old man with bilateral THA and with the longest latent period from the performed THA to established diagnosis reported so far.

Given the large number of total hip arthroplasties that are routinely performed all over the world, the development of a malignant lesion appears to be an extraordinary complication and may be just a coincidence or the result of some derangement of the healing process in host tissue with no definitely proven hypothesis that the implants or their by-products are carcinogenic. The very low number of confirmed cases of implant-related tumors in humans makes such a hypothesis very uncertain.

The importance of our presented case is to alert orthopedic surgeons to the clinical, radiologic, and macroscopic similarities between a malignant tumor and benign lesions caused by wear debris at THA sites. At the examination of plane X-rays of patients with THA loosening, the differential diagnosis should always include osteogenic sarcoma, as well.

## Consent

“Written informed consent was obtained from the patient for publication of this Case report and any accompanying images.

## Abbreviations

THA: total hip arthroplasty
ROM: range of motion
OA: osteoarthritis
AS: ankylosing spondylitis
PE: polyethylene
ND: no data
